# Biventricular Thrombi Associated with Peripartum Cardiomyopathy

**DOI:** 10.3329/jhpn.v29i2.7862

**Published:** 2011-04

**Authors:** Dong-Yeon Kim, Saidul Islam, Nelson Taposh Mondal, Felicity Mussell, Matthea Rauchholz

**Affiliations:** ^1^Department of Internal Medicine; ^2^Department of Obstetrics and Gynecology, LAMB Hospital, Rajabashor, Parbatipur, Dinajpur, Bangladesh

**Keywords:** Biventricular thrombi, Case studies, Peripartum cardiomyopathy, Bangladesh

## Abstract

A 22-year old woman visited the LAMB Hospital, Parbatipur, Dinajpur, Bangladesh, in February 2010, with exertional dyspnea for three weeks. She had had a normal vaginal delivery four months ago; 2-dimensional echocardiogram showed severe left ventricular dysfunction and biventricular thrombi, which resolved without complications after anticoagulation. Biventricular thrombosis with peripartum cardiomyopathy is quite a rare finding, and its clinical course and proper management is not known. No such case has previously been reported in Bangladesh.

## INTRODUCTION

Peripartum cardiomyopathy (PPCM) is a rare cardiac disorder which leads to heart failure in the last month of pregnancy or up to five months postpartum (1). PPCM is associated with a hypercoagulative state, which can cause thromboembolic complications, such as intracardiac thrombus detected by 2-dimensional echocardiogram. The clinical course and proper management of intracardiac thrombus with PPCM is yet to be elucidated. We report a case of biventricular thrombosis with PPCM, which was resolved with anticoagulation treatment without embolic complications. To the best of our knowledge, this is the first report in Bangladesh.

## CASE REPORT

**Fig. 1.**
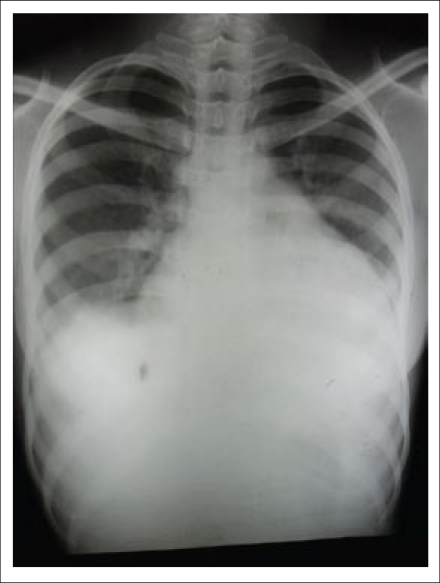
Chest x-ray showing enlarged cardiac shadow and bilateral pulmonary oedema

**Fig. 2.**
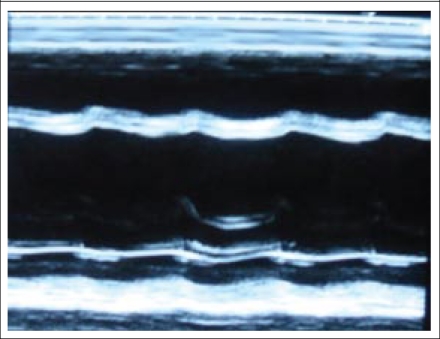
M-mode echocardiogram showing dilated left ventricle and reduced left ventricle systolic function

**Fig. 3.**
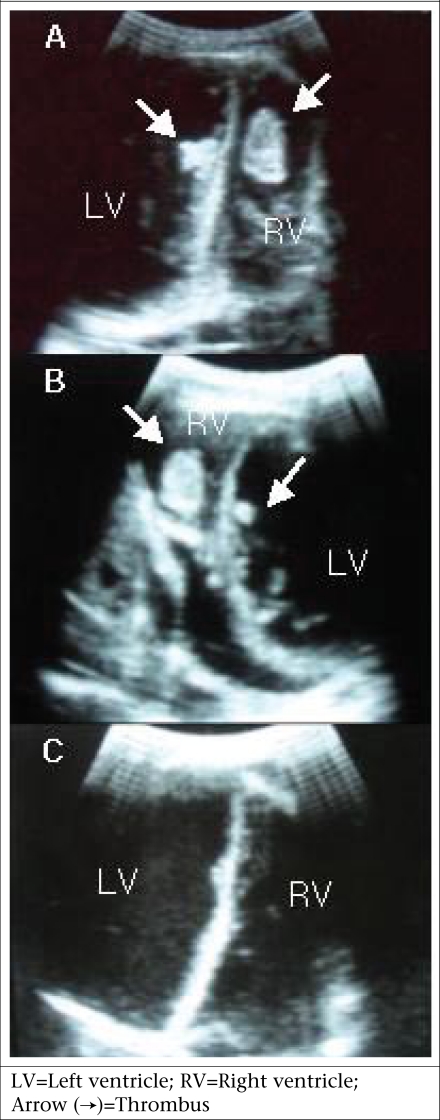
Echocardiogram showing biventricular thrombi (A, B), and the thrombi had disappeared on the 21st day after anticoagulation (C)

A 22-year-old woman visited the LAMB Hospital, Parbatipur, Dinajpur, Bangladesh, in February 2010, complaining of general weakness and exertional dyspnea for three weeks. She had had a normal vaginal delivery at home four months ago and denied any specific history of medical illness. Her blood pressure was 90/60 mm Hg, pulse rate 120 beat/min, respiration rate 24/min, and body temperature 37.2 °C. The heart-beat was regular without any audible cardiac murmur. Fine rales were heard at both basal lung fields. Chest x-ray showed cardiomegaly with bilateral pulmonary congestion (Fig. 1). Electrocardiogram showed sinus tachycardia. Her haemoglobin was 12.0 g/dL, with total white cell count of 8,000/mm^3^. The platelet count was normal (240,000/mm^3^) with normal prothrombin time (15 seconds). Her liver enzyme was mildly elevated (serum aspartate transaminase–126.7 IU/L) while her serum creatinine was normal (86.5 µmol/L). A 2-dimensional and M-mode echocardiogram showed that both the ventricles were enlarged and that the left ventricular systolic function was depressed (Fig. 2). The left ventricular end-diastolic dimension was 64 mm, and the ejection fraction was 17%. Intracardiac thrombi were noted in both left and right ventricles (Fig. 3).

Enalapril (5 mg) and furosemide (80 mg) were given. Warfarin (5 mg) was started in combination with subcutaneous heparin injection.

On the 16th day of admission, the follow-up echocardiogram showed no thrombus in the left ventricle while the large thrombus in the right ventricle was still visible, unchanged in size. Her pulmonary congestion and clinical symptoms had improved despite the large thrombus in the right ventricle. She was discharged on her request on the same day. The dose of warfarin was adjusted to 2.5 mg daily, as the prothrombin time was prolonged up to 65 seconds (international normalized ratio=5.0) without any bleeding complications.

On the 21st day, she visited the clinic for repeat echocardiogram. There was no visible intracardiac thrombus in either ventricle (Fig. 3). She came back to the clinic two months later without thromboembolic complications on anticoagulation with warfarin. The follow-up echocardiogram showed no intraventricular thrombus, although the left ventricular systolic function remained depressed.

## DISCUSSION

PPCM is a disease of uncertain aetiology, characterized by left ventricular systolic dysfunction and symptoms of heart failure. It occurs in previously-healthy women mainly at the end of pregnancy and up to five months after delivery (1). The incidence of PPCM has been estimated to range from one case per 2,289 to one case per 4,000 livebirths in the USA, one case per 299 livebirths in Haiti, and one case per 1,000 livebirths in South Africa (2). The reason for such a variation in incidence among different countries remains unknown.

Ventricular thrombus, a potentially life-threatening condition, is frequently detected by echocardiogram in PPCM patients with an ejection fraction of less than 35% (2). Patients with PPCM already face an increased risk of thromboembolism because of the procoagulant activity postpartum due to the elevation of factors VII, X, VIII, fibrinogen, and von Willebrand factor (3). Severe biventricular dysfunction, in addition to a hypercoagulable state during pregnancy, may increase the risk of ventricular thrombosis in patients with PPCM (4).

The proper management for intraventricular thrombus with PPCM is still to be elucidated. A few case reports showed that heparinization was effective, particularly with fresh thrombus; 3-5 days were taken for thrombi to dissolve (5). Recent thrombi are more shaggy and irregular in configuration whereas organized thrombi are more circumscribed and immobile. Fresh thrombi also have a low echoic shadow and are non-laminar. The thrombi of our case seemed organized, less mobile, and with a regular surface on echocardiogram. Anticoagulation therapy with heparin and warfarin was effective in resolving the organized biventricular thrombi in our patient, although it took about three weeks for full resolution of the thrombi. The use of thrombolytic agents or embolectomy to treat intraventricular thrombi could be justified because of fear of embolic complications, although there is no consensus for patients with PPCM and ventricular thrombosis so far.

The clinical course of PPCM varies, with 50-60% of patients showing complete or near-complete recovery of clinical status and cardiac function, usually within the first six months postpartum; the remaining patients demonstrate either further clinical deterioration, leading to cardiac transplantation or premature death, or persistent left ventricular dysfunction and chronic heart failure (6). Eventual recovery of left ventricular systolic function occurs more frequently in women who had an ejection fraction of greater than 30% at original diagnosis of PPCM (2). Our patient had a very low ejection fraction without improvement in the first two months, which is associated with poor long-term outcome. Since a low ejection fraction predicts a higher risk of thromboembolic complications, we planned to continue anticoagulation until the left ventricular function becomes normal in our case.

Family-planning counselling is an important aspect of the care of patients after a diagnosis of PPCM. Subsequent pregnancy after a diagnosis of PPCM carries a higher risk of relapse if left ventricular systolic function has not fully recovered first, and even with full recovery, some additional risk of relapse remains (2). Physicians and obs-tetricians should advise an appropriate method of family planning for all patients with PPCM. Since combined oral contraceptives increase the risk of thromboembolism, their use should be discouraged in this setting. Injectable contraceptives are not feasible when patients need active anticoagulation. Either subdermal implants or an intrauterine device can be an option, if male sterilization is culturally unacceptable.
